# Genomic signatures of adaptation to Sahelian and Soudanian climates in sorghum landraces of Senegal

**DOI:** 10.1002/ece3.5187

**Published:** 2019-04-23

**Authors:** Jacques M. Faye, Fanna Maina, Zhenbin Hu, Daniel Fonceka, Ndiaga Cisse, Geoffrey P. Morris

**Affiliations:** ^1^ Department of Agronomy Kansas State University Manhattan Kansas; ^2^ Institut National de la Recherche Agronomique du Niger Niamey Niger; ^3^ Centre d'Étude Régional pour l'Amélioration de l'Adaptation à la Sécheresse Thiès Sénégal; ^4^ CIRAD UMR AGAP Montpellier France

**Keywords:** Africa, drought tolerance, local adaptation, selective sweeps, stay‐green

## Abstract

Uncovering the genomic basis of climate adaptation in traditional crop varieties can provide insight into plant evolution and facilitate breeding for climate resilience. In the African cereal sorghum (*Sorghum bicolor* L. [Moench]), the genomic basis of adaptation to the semiarid Sahelian zone versus the subhumid Soudanian zone is largely unknown. To address this issue, we characterized a large panel of 421 georeferenced sorghum landrace accessions from Senegal and adjacent locations at 213,916 single‐nucleotide polymorphisms (SNPs) using genotyping‐by‐sequencing. Seven subpopulations distributed along the north‐south precipitation gradient were identified. Redundancy analysis found that climate variables explained up to 8% of SNP variation, with climate collinear with space explaining most of this variation (6%). Genome scans of nucleotide diversity suggest positive selection on chromosome 2, 4, 5, 7, and 10 in durra sorghums, with successive adaptation during diffusion along the Sahel. Putative selective sweeps were identified, several of which colocalize with stay‐green drought tolerance (*Stg*) loci, and a priori candidate genes for photoperiodic flowering and inflorescence morphology. Genome‐wide association studies of photoperiod sensitivity and panicle compactness identified 35 and 13 associations that colocalize with a priori candidate genes, respectively. Climate‐associated SNPs colocalize with *Stg3a*, *Stg1*, *Stg2*, and *Ma6* and have allelic distribution consistent with adaptation across Sahelian and Soudanian zones. Taken together, the findings suggest an oligogenic basis of adaptation to Sahelian versus Soudanian climates, underpinned by variation in conserved floral regulatory pathways and other systems that are less understood in cereals.

## 1 INTRODUCTION

Local adaptation is critical for survival of traditional crop varieties in stressful environments (Camus‐Kulandaivelu et al., 2006; Xu et al., 2006). Smallholder farmers in developing countries are particularly vulnerable to environmental factors such as drought and heat stress limiting crop production (Morton, 2007). Climatic gradients in relation to precipitation are major drivers of adaptation in plants including traditional crop varieties (Fournier‐Level et al., 2011; Lasky et al., 2015, 2012; Siepielski et al., 2017; Vigouroux et al., 2011). Adaptation to water‐limited environments involves phenological, physiological, and morphological traits such as photoperiod sensitivity, delayed senescence, and inflorescence morphology (Blum, 2014). For instance, when growing seasons are shortened by end‐of‐season droughts, selection favors early maturity alleles to escape drought (Franks, Sim, & Weis, 2007; Kenney, McKay, Richards, & Juenger, 2014; Vigouroux et al., 2011). Identifying genetic polymorphisms underlying adaptive traits and their eco‐geographic distributions is necessary to understand the genetic basis of local adaptation of landraces (Romero Navarro et al., 2017).

The patterns of genome‐wide nucleotide polymorphisms provide insight into selective forces varying over time and space (Olsen et al., 2006; Slatkin, 2008). Recent studies in rice (Caicedo et al., 2007; Li, Li, Jia, Caicedo, & Olsen, 2017), tomato (Lin et al., 2014), and maize (Swarts et al., 2017) have shown that high genetic differentiation among populations reflects adaptation to specific agroclimatic zones. Population genomic approaches for identifying signatures of selection include decreased pairwise nucleotide diversity, composite likelihood ratio (CLR) analysis for selective sweeps, and genome–environment associations (GEA) (Fang et al., 2017; Fournier‐Level et al., 2011; Lasky et al., 2015; Li et al., 2017; Lin et al., 2014). The CLR analysis in SweeD is relatively robust to demographic events because the method conservatively estimates the neutral site frequency spectrum (SFS) based on the observed data (Nielsen et al., 2005; Pavlidis, Živković, Stamatakis, & Alachiotis, 2013). Linear regression models and genome‐wide association studies (GWAS) mixed models are common methods used for GEA, especially to investigate adaptation to environmental gradients (Rellstab, Gugerli, Eckert, Hancock, & Holderegger, 2015), and have been applied by several studies in plants and crop species (Fournier‐Level et al., 2011; Lasky et al., 2015; Yoder et al., 2014). Redundancy analysis (RDA) provides an estimate of allelic variance explained by climatic factors based on multivariate linear regressions (Meirmans, 2015). Genome‐wide association studies can provide high mapping resolution of adaptive traits in diverse populations (Cavanagh, Morell, Mackay, & Powell, 2008).

Sorghum (*Sorghum bicolor* L. [Moench]) is a staple food crop for smallholder farmers in semiarid regions worldwide. The modest genome size (~800 Mbp) of sorghum relative to other grass species (Paterson et al., 2009) makes it a tractable system for the genomic studies of local adaptation. Five botanical types (bicolor, durra, guinea, caudatum, and kafir) have been described (Harlan & De Wet, 1972). Durra types, known for their adaptation to arid zones, are thought to have originated in Ethiopia before westward diffusion along the Sahel to West Africa and finally Senegal (Harlan & De Wet, 1972). Guinea types, known for their humid adaptation (Deu et al., 1994; Folkertsma, Rattunde, Chandra, Raju, & Hash, 2005), may reflect a second center of domestication in the humid savanna of West Africa (Deu et al., 1994; Doggett, ; Folkertsma et al., 2005). Inflorescence morphology is a major component of agroclimatic adaptation in sorghum and varies from loose panicle in guinea to compact panicle in durra sorghum (Brown et al., 2006). Most traditional sorghum varieties in West Africa are photoperiod sensitive such that grain maturation coincides with the end of the rainy season (Bhosale et al., 2012; Sanon et al., 2014). In U.S. sorghum, variation in flowering time is controlled by conserved cereal floral regulatory networks, including phytochromes (*Ma3/PhyB*, *Ma5/PhyC*), CCT‐domain regulators (*Ma1/PRR37*, *SbEhd1, SbEhd2)*, and florigens (*SbCN15*/*Hd3a*, *SbCN12*) (Mullet et al., 2010; Murphy et al., 2011). Several quantitative trait loci (QTL) (*Stg1–4*) confer stay‐green (i.e., delayed leaf senescence) postflowering drought tolerance in lines derived from Ethiopian durra (Borrell et al., 2014; Harris et al., 2007; Kebede, Subudhi, Rosenow, & Nguyen, 2001; Tuinstra, Grote, Goldsbrough, & Ejeta, 1997), but it is not known whether these loci contribute to drought adaptation more widely across the Sahel.

Analyses of genetic diversity, linkage disequilibrium (LD), and GEA have provided an understanding of worldwide sorghum genetic structure across diverse agroclimatic regions (Bouchet et al., 2012; Lasky et al., 2015; Mace et al., 2013; Morris et al., 2013; Wang et al., 2013). However, the genomic basis of climate adaptation at a regional scale remains poorly understood. The variation of agroclimatic conditions in Senegal reflects the sub‐Saharan climatic gradient with increasing annual precipitation from north to south across the Sahelian (~200–600 mm) and Soudanian zones (~600–1100 mm). A large panel of sorghum landraces was collected from these agroclimatic zones in Senegal in the 1970s (Clément & Houdiard, 1977). To better understand the genomic basis of Sahelian and Soudanian climate adaptation, we used genotyping‐by‐sequencing (GBS) to characterize genome‐wide single‐nucleotide polymorphism (SNP) in georeferenced and phenotyped Senegalese sorghum landraces. We characterized population structure of genomic diversity, identified signatures of selection, and mapped genetic polymorphisms associated with phenotype and climate. The findings suggest that climate has shaped genomic variation across Sahelian and Soudanian zones, with variation in floral regulatory pathways and other systems contributing to this adaptation.

## 2 MATERIALS AND METHODS

### 2.1 Plant materials

The Senegalese sorghum germplasm (SSG) used in the present study were obtained from the U.S. Department of Agriculture (USDA) Germplasm Resources Information Network (GRIN). These accessions (*n* = 341) were collected from various agro‐ecological zones of Senegal in 1976 (Clément & Houdiard, 1977). Germplasm Resources Information Network accessions from neighboring countries of Gambia (*n* = 60), which is surrounded by Senegal, and Mauritania (*n* = 15), which shares border along the Senegal River Valley, were also included in our panel. Six improved varieties (CE 151‐262, CE 180‐33, ISRA‐S‐621‐B, 53‐49, CE 260‐12‐1‐1, IRAT 4) from the sorghum breeding program based at the Centre National de Recherche Agricole (CNRA) and two sorghum conversion lines, SC 1,067 (PI 576,432) and SC 417 (PI 533,861), were included. Information about the SSG including botanical race, geographic origin, local name, and ethno‐linguistic group from which the landrace was collected are presented in Data S1. Assignment in “durra” group was from the GRIN genebank, based on a phenotypic assessment. To compare the SSG landraces with the global sorghum diversity, we reanalyzed available raw sequencing data of worldwide sorghum diversity panels (Morris et al., 2013), hereafter referred to as the global diversity panel (GDP). This data set included 582 lines from the sorghum mini core collection and the Generation Challenge Program reference set, and 178 lines from the sorghum association panel. The GDP includes accessions from Africa, Asia, and the Americas.

### 2.2 Genotyping‐by‐sequencing

Accessions of the SSG were grown in a glasshouse at Kansas State University. Leaf tissues from each accession were harvested from two weeks old seedlings (five seedlings pooled per accession), placed into 96‐well plates, and dried in a lyophilizer for two days. Genomic DNA of SSG accessions was extracted from ~50 mg dried leaf tissue using the BioSprint robot with DNeasy Mini Kit (Qiagen) according to the manufacturer's instructions. DNA was quantified with PicoGreen and normalized to 10 ng/μl DNA for each sample. The GBS library was constructed using the restriction enzyme *Ape*KI for DNA digestion and 384‐plex barcode ligation (4 × 96‐plex) following the GBS protocol (Elshire et al., 2011). Digested DNA fragments were ligated to the barcode‐adapters in a solution containing the 10× T4 DNA Ligase Reaction Buffer, ultrapure water, and T4 DNA Ligase (New England Biolabs), then cleaned using a QIAquick PCR purification kit (Qiagen). The adapter‐ligated DNA fragments were amplified by polymerase chain reaction (PCR). The PCR‐amplified DNA fragments were cleaned and quantified with PicoGreen. Four 96‐plex libraries were pooled to form a 384‐plex GBS library. GBS libraries were diluted into 20 μl at 4 nM for each library and analyzed by the Agilent 2100 Bio‐analyzer for sequencing. GBS libraries were sequenced on Illumina HiSeq 2500 at the University of Kansas Medical Center.

### 2.3 SNP calling

The SNP calling was done based on 1,208 samples including the accessions from the SSG panel and accessions from the GDP. Single‐end sequence reads obtained from Illumina sequencing and raw sequencing data from the GDP were processed with the TASSEL 5 GBS v2 pipeline (Glaubitz et al., 2014). All unique sequence reads were trimmed to 64 bp, which was the default setting. The first step in the pipeline (GBSSeqToTagDBPlugin) allowed to collapse identical reads into tags using the key files of both SSG and GDP accessions. Distinct tags were pulled and exported from the database in the FASTQ format using the TagExportToFastqPlugin for their alignment to the BTx623 sorghum reference genome v.3.1 (McCormick et al., 2018; Paterson et al., 2009). The alignment was performed with the Burrows–Wheeler Alignment (Li & Durbin, 2009) where the created SAM file was passed through the SAMToGBSdbPlugin to store the position information of aligned tags. The SNPs were called from the aligned tags. The DiscoverySNPCallerPlugin was used to identify SNPs from the aligned tags where minor allele frequencies (MAF) was set to 0.0001 and minimum locus coverage (mnLCov) was kept as the default setting of 0.1. For downstream population genomic analyses, SNPs with <20% missing data rate and MAF > 0.01 were retained. Monomorphic sites were removed and only biallelic sites were retained. Missing genotypes were imputed using Beagle v4.1 program (Browning & Browning, 2016). For the association mapping studies, the SNP data set was filtered for MAF > 0.05 to reduce the chance of observing false‐positive associations.

### 2.4 Population structure analysis

Principal components analysis (PCA) of SNP variation was performed using the snpgdsPCA function of the R package SNPRelate (Zheng et al., 2012). Neighbor‐joining (NJ) analysis was performed using TASSEL 5 program, and the tree was visualized with the ape package in R (Paradis, Claude, & Strimmer, 2004). Bayesian model‐based clustering in ADMIXTURE v1.23 (Alexander, Novembre, & Lange, 2009) was used to estimate the subpopulation membership/admixture for *K* = 2–20 subpopulations. To reduce SNP redundancy due to LD for the admixture analysis, genotypic data were LD‐pruned with a *window size* of 50 SNPs, *step size* 10, and *VIF threshold* of 0.5 using the function *indep* in PLINK 1.9 (Purcell et al., 2007). Default settings of ADMIXTURE were used, and fivefold cross validation (CV) error with block bootstrap and 2,000 iterations was used to determine the optimum value of *K*. Each accession was assigned to subpopulation when the proportion of the coefficient of membership to subpopulation was >0.60. To determine the spatial genetic co‐ancestry structure with respect to geography, we used the R package TESS3 (Caye, Deist, Martins, Michel, & François, 2016). Results were visualized using the R program (R Core Team, 2016).

### 2.5 Linkage disequilibrium analysis

LD was characterized in the whole SSG and separately in the guinea and durra accessions. VCFtools (Danecek et al., 2011) was used to filter the genotypic data based on MAF > 0.05. The pairwise correlation coefficient (*r* ^2^) among SNPs was used to estimate LD using TASSEL 5 (Bradbury et al., 2007). LD decay, measured as the distance by which the *r* ^2^ decays to half its maximum value, was fit using the nonlinear least square (*nls*) function (Hill & Weir, 1988; Remington et al., 2001) in R program. The R package LDheatmap 0.99‐4 (Shin, Blay, McNeney, & Graham, 2006) was used to determine and display the pairwise LD surrounding (50 kb region from both sides of the SNP) a SNP‐environment variable association.

### 2.6 Genome‐wide nucleotide variation and genome scans

Minor allele frequencies and observed and expected heterozygosity for SNP markers were calculated using VCFtools program and R program (R Core Team, 2016). Pairwise genetic differentiation (*F*_ST_) among subgroups defined based on eco‐geography was estimated using the Weir and Cockerham method in VCFtools. *F*_ST_ values among subgroups obtained at *K* = 7 from the TESS3 program were calculated using the R package HierFstat (de Meeûs & Goudet, 2007). Pairwise genome‐wide nucleotide diversity (π) and Tajima's *D* test statistics were calculated based on nonoverlapping sliding windows of 1 Mbp across the genome using VCFtools. Ratios of *π* were analyzed between guinea and durra accessions in the SSG (*π*_guinea_/*π*_durra_), and across putative prebottleneck and postbottleneck events (*π*_guinea_/*π*_Ethiopia durra_, *π*_Ethiopia durra_/*π*_Niger and Mali durra_, and *π*_Niger and Mali durra_/*π*_Senegal durra_). Selective sweeps were detected using the CLR method in SweeD program (Pavlidis et al., 2013). Each chromosome was divided into 5,000 grid points (nonoverlapping windows). The CLR windows with ≥8 SNPs (approximately 1 SNP per 2 kb) were retained during the analysis. The significance threshold representing the 95th percentile cutoff was determined based on 1,000 simulations.

### 2.7 Genome‐wide association studies (GWAS)

GWAS were carried out using mixed‐linear models (MLM) in GAPIT in R (Lipka et al., 2012) with the three first principal components eigenvectors and kinship matrix. The Bonferroni correction at *α* = 0.05 level was used to define the significance of association tests. SNPs were filtered at MAF > 0.05, yielding 145,235 SNPs. Phenotypic data were obtained from the GRIN database and treated as binary data for both photoperiod sensitivity (e.g., sensitive vs. insensitive) and panicle compactness (e.g., compact vs. open panicle). For GEA, both MLM and general linear models (GLM) were used. Nineteen WorldClim‐derived bioclimatic variables (Hijmans, Cameron, Parra, Jones, & Jarvis, 2005) were used for genome–environment association tests. To identify environment‐associated SNPs with the greatest significance among SNPs of the same genomic region, the multilocus mixed‐model (MLMM) (Segura et al., 2012) was used to complement the GLM and MLM. In both MLM and MLMM, the first three principal components were included to account for population structure.

### 2.8 A priori candidate genes

A list of a priori candidate genes for climate adaptation was defined from known sorghum genes, orthologs of cloned genes from rice and maize, and candidates from previous sorghum mapping studies (see Data S2 for candidate genes, gene functions, and references). A literature survey of sorghum orthologs of maize and rice genes that affect inflorescence architecture, flowering time, and drought tolerance was carried out. Inflorescence architecture candidate genes from a previous global GWAS (Morris et al., 2013), photoperiodic flowering time candidate genes from a previous study (Bhosale et al., 2012), and validated drought tolerance loci (stay‐green, *Stg1–4*) from (Borrell et al., 2014) were included. Genomic position of candidate genes was determined using Phytozome v12.1.6 (https://phytozome.jgi.doe.gov) (Goodstein et al., 2012).

### 2.9 Redundancy analysis

RDA was performed using the R package vegan (Oksanen et al., 2017) for climatic factors, ethnicity, and space. Independent variables included nineteen climatic variables, space variables (latitude and longitude), and ethnicity variables. Ethnicity was coded as binary variable indicating the ethno‐linguistic group of the farmer that contributed the landrace to the collection (Clément & Houdiard, 1977). Forward selection based on 1,000 permutations was performed for space (e.g., using polynomial coordinates), climate, and ethnicity variables to include only the meaningful variables for ordination. The total among‐population genetic variance was partitioned into space, climate, ethnicity, and their overlapping fractions using 1,000 randomly selected SNP (MAF > 0.05). The significance of each variance fraction was tested with 1,000 permutations.

## 3 RESULTS

### 3.1 Genome‐wide SNP variation in Senegalese sorghum

**Figure 1:**
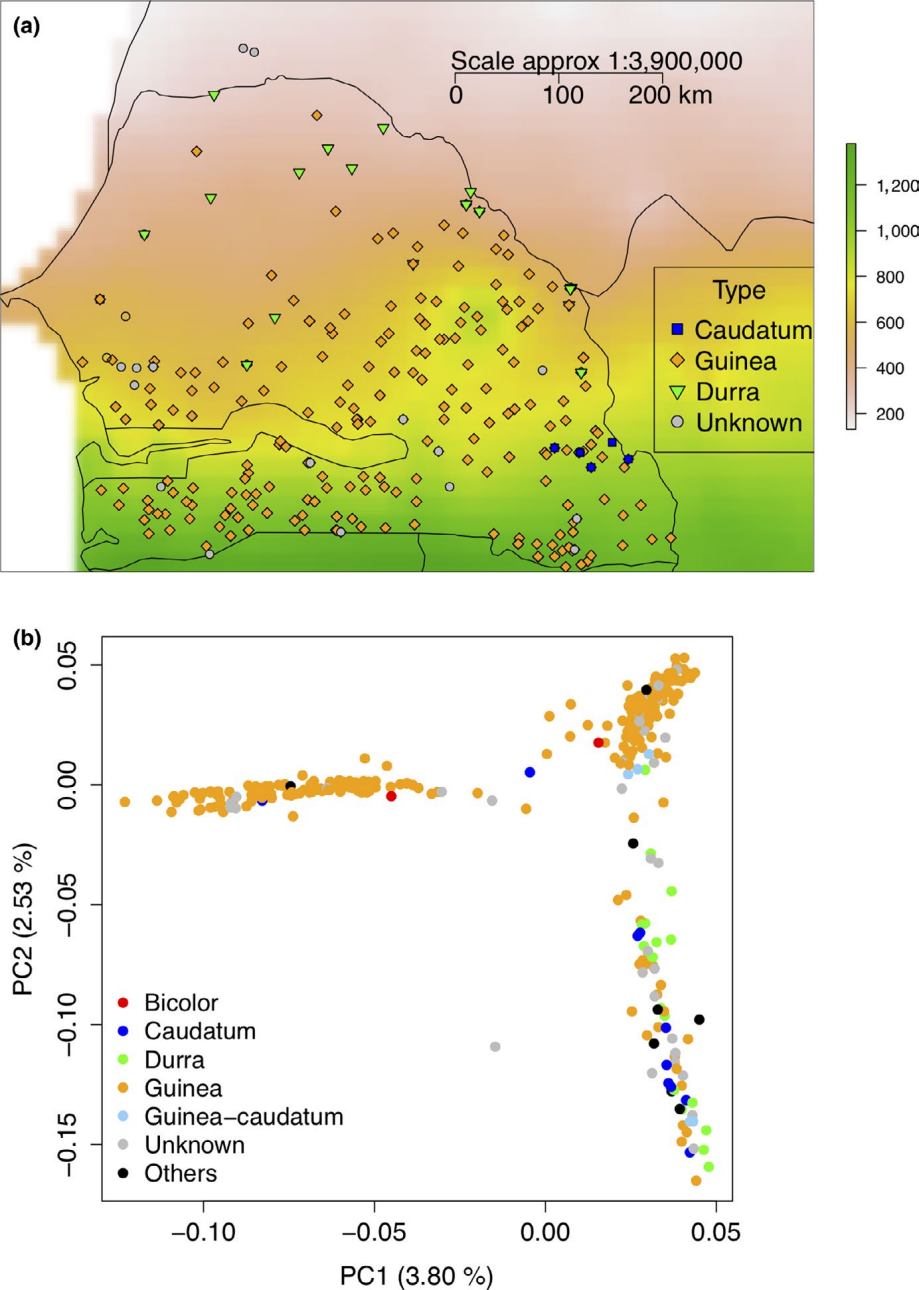
SNP variation in the Senegalese sorghums accessions. (a) Geographic distribution of the Senegalese sorghums accessions along precipitation gradient. The accessions are colored coded with respect to botanical race. The color background scale indicates the annual precipitation in millimeters with green color representing the highest precipitation of the Soudanian zone, pink representing lowest precipitation of the Sahelian zone, and yellow representing the zone of transition between Sahelian and Soudanian zones. (b) Scatterplot of the two first principal components explaining the genomic variation within the SSG collection

The Senegalese sorghum accessions included in this study originated from diverse agroclimatic zones (Figure 1a), agro‐ecological regions (Figure S1a), and ethnic–linguistic groups (Figure S1b). Across 421 accessions, we identified 213,916 SNPs after filtering out SNPs with >20% missing data, MAF < 0.01, and retaining only biallelic SNPs. The SNP density was determined based on nonoverlapping windows of 1 Mb where SNPs were distributed across the genome with higher density in the subtelomeric regions (Figure S2a). The SNPs covered most of the genome with an average coverage of 1 SNP every 2 kb. The average observed and expected heterozygosity in the SSG were estimated at 0.05 and 0.23, respectively. The average pairwise nucleotide diversity (*π*) was 0.00054 in durra and 0.00060 in guinea accessions. The average pairwise LD (*r* ^2^) decreased from its initial value (~0.5) to 0.2 at 220 kb, 150 kb, and 81 kb in durra, guinea, and whole SSG, respectively (Figure S2b). LD decayed to background level (~0.1) at 880 kb in durra and 430 kb in guinea. The SSG had a lesser proportion of low frequency minor alleles (<5% MAF) and greater proportion of intermediate frequency minor alleles than the GDP, based on nonoverlapping window size of 1 Mb (Figure S2c). About 60% of SNPs were rare (MAF < 0.05).

Next, we investigated the genetic variation and structure of the SSG. The two first principal components explained 3.8% and 2.5% of SNP variation (Figure 1b). The accessions originated from the center formed one cluster, accessions from the south formed a second cluster, and accessions from the north formed a third cluster. The third cluster included durra accessions, caudatum accessions, a few guinea accessions from the north, and improved varieties. Neighbor‐joining tree matched the PCA results and revealed that SSG durra accessions were closely related to the durra from Ethiopia and other West African countries (Figure S2d). Durra and guinea accessions within the SSG were genetically differentiated from each other. The SSG also clustered somewhat with respect to ethno‐linguistic groups, which are nested within geographic origins of the accessions (Figure S2e).

### 3.2 Model‐based population structure and variance partitioning

**Figure 2:**
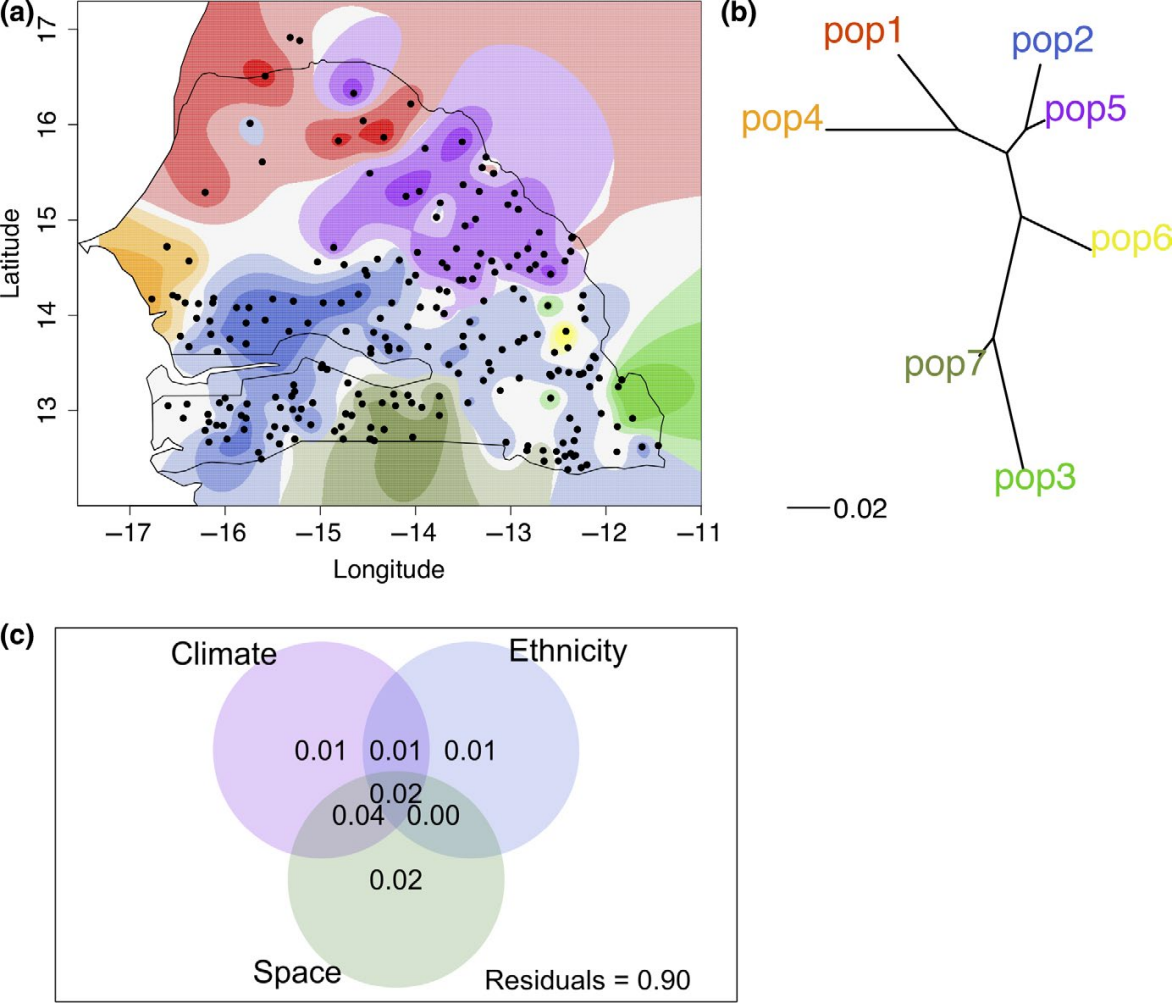
Spatial population structure and SNP variance partitioning in the Senegalese sorghum. (a) Spatial genetic co‐ancestry structure of the accessions at *K* = 7. Each accession is represented by dot on the map and each color represents a genetic co‐ancestry matrix. (b) The *F*_ST_ genetic differentiation among subpopulations at *K* = 7 ancestral groups from b; the color‐coding matches that in a. (c) Among‐population genetic variance at 1,000 randomly selected SNPs with MAF > 0.05 explained independently by climatic, space, and ethnicity variables

To further characterize genetic structure and gene flow among groups, we used Bayesian model‐based clustering. ADMIXTURE revealed a hierarchical genetic structure and high amount of gene flow among subpopulations (File S1). Cross validation error was minimized with *K* = 7 subpopulations (Figure S3). We investigated the spatial genetic co‐ancestry in the SSG with TESS3 based on allele frequency distribution and geographic origin. Seven optimum spatial genetic clusters (*K* = 7) were identified (Figure 2a). The TESS3 results matched the ADMIXTURE groups for different *K* values. Genetic differentiation among the subpopulations (including only samples with admixture rate ≥ 0.7) found at *K* = 7 from TESS3 results was determined using the *F*_ST_ analysis (Figure 2b). The durra accessions from the northern subpopulation (pop1) were more related to the improved varieties (pop4), based on *F*_ST_ analysis. Both pop1 and pop4 were distinct from central and southern subpopulations (pop2, 3, 5–7), which were mostly formed by guinea accessions where guineas in the center were differentiated from guineas in the south. *F*_ST_ of 0.185 and 0.052 were estimated between guinea and durra accessions in the SSG, and between SSG durra and GDP durra, respectively.

We used RDA to estimate the proportion on SNP variation explained by climate variation, ethno‐linguistic origin, and space. Climate and ethnicity explained up to 6% and 4% of SNP variance, respectively, including variance collinear with space (*p* > 0.001) (Figure 2c). After accounting for space, climate and ethnicity explain up to 2% of the variance, each. Climate collinear with space, the putative proportion of clinal adaptation, explained 6% of variance.

### 3.3 Genome‐wide patterns of nucleotide polymorphism

**Figure 3:**
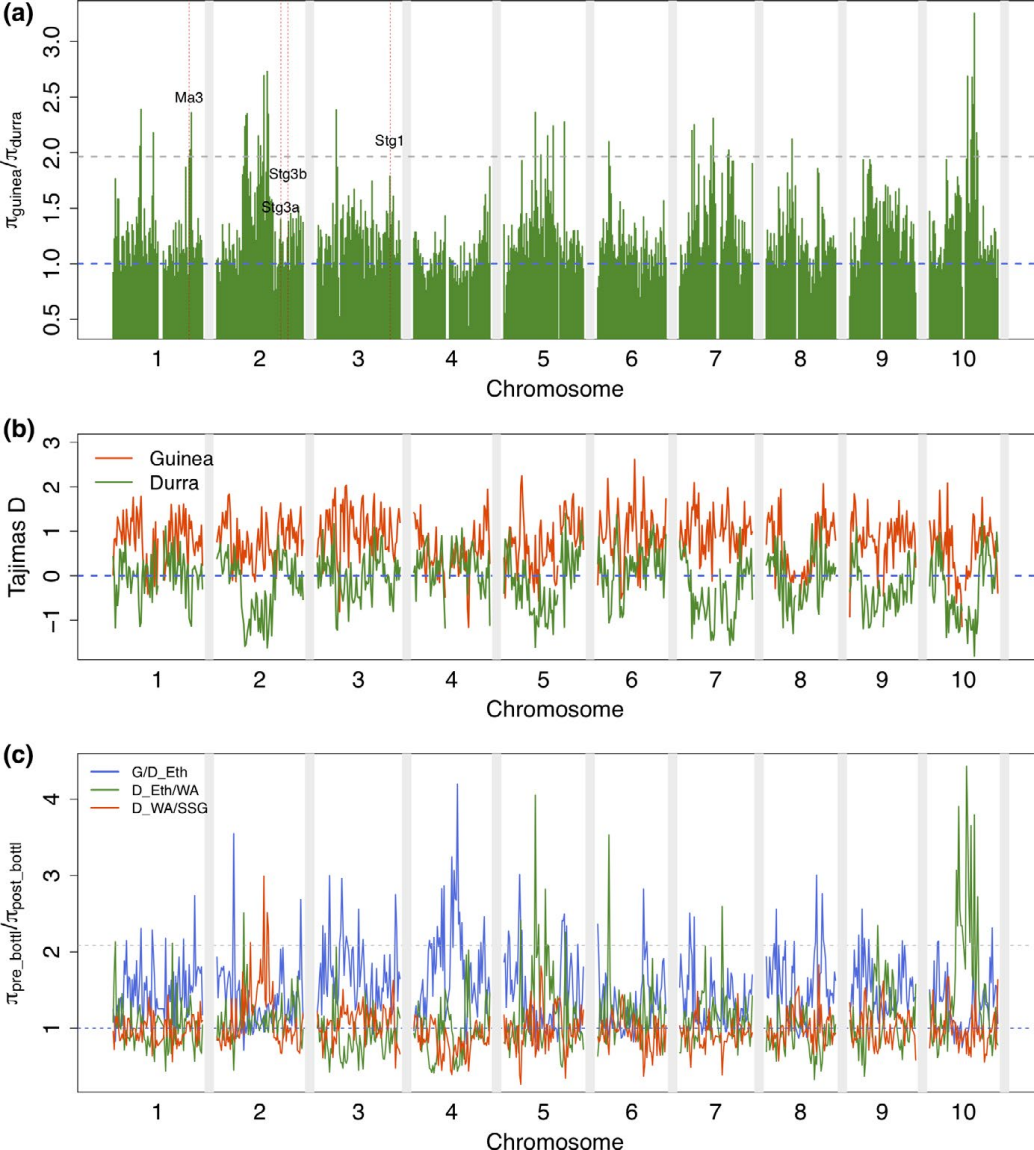
Genome‐wide pattern of nucleotide diversity in durra accessions. Decrease in pairwise nucleotide diversity and Tajima's *D* test for nonoverlapping sliding windows of 1 Mbp across the genome. (a) Decreased pairwise nucleotide diversity in durra relative to guinea in the Senegalese sorghum. The horizontal dashed lines indicate the mean value (blue) and the top 5% (gray) of decreased nucleotide diversity. (b) Tajima's *D* test between durra (green) and guinea (red) accessions in Senegalese sorghum. (c) Positive selections between durra from Ethiopia and all guineas in the global diversity panel (blue), between Ethiopian durra and West African durra (green), and between West African durra and Senegalese durra (red)

To identify genomic regions subject to selection, we compared genome‐wide nucleotide polymorphism (*π*) between guinea (Soudanian) and durra (Sahelian) accessions within the SSG. Since guinea sorghums are generally more genetically diverse than durra sorghums, we used *π*_guinea_ in the numerator and *π*_durra_ in the denominator to identify low‐diversity genomic regions in the durra genome. Nucleotide polymorphism was reduced in durra compared to guinea across most of the genome, with notably low *π* on pericentromeric regions of chromosome 2, 5, 7, and 10 (Figure 3a). For durra, 34 genomic regions (1 Mb windows) were identified as putative selected regions (top 5% cutoff > 1.96). A notable region of low *π*_durra_ on chromosome 1 colocalized with the *Ma3* photoperiodic flowering gene. Modestly lower *π*_durra_ was observed around *Stg1*, *Stg3a*, and *Stg3b*. Generally, negative values of Tajima's *D* were observed in durra, contrasting the positive values observed in guinea (Figure 3b).

To better understand the timing of putative selection events, we investigated ratios of nucleotide polymorphism across three putative genetic bottlenecks: (a) since the divergence of durra from its common ancestor with guinea types, (b) from Ethiopian durra (center of durra origin) to West African durra (Niger and Mali), (c) and from West African durra to Senegalese durra (Figure 3c). We also characterized nucleotide polymorphism between all Sahelian durra against worldwide guinea (Figure S4). The *π* reduction in the pericentromeric regions of chromosome 4 occurred mainly in Ethiopian durra. The *π* reduction on pericentromeric regions of chromosomes 5 and 10 and subtelomeric region of chromosome 6 were common to all West African durra sorghums. The *π* reduction in the pericentromeric region of chromosome 2 was specific to the SSG durra.

### 3.4 Selective sweeps and colocalization of a priori candidate genes

**Figure 4:**
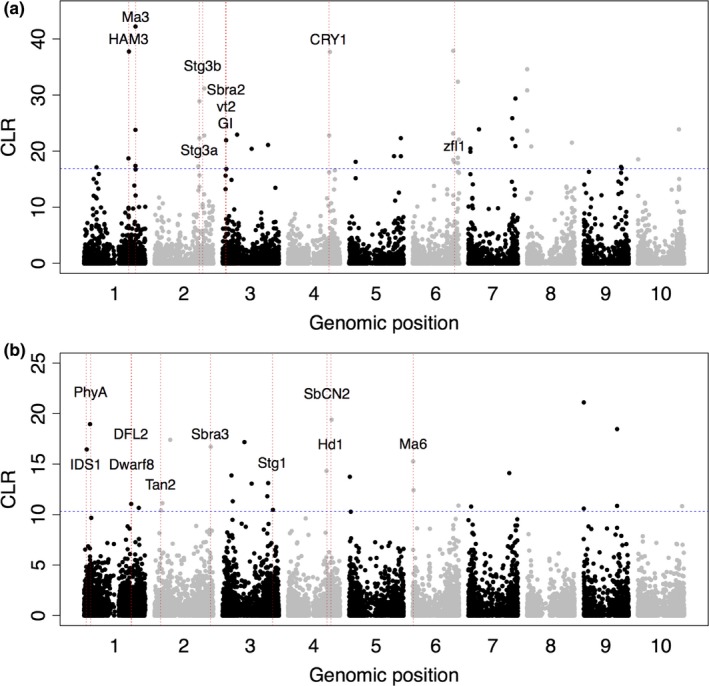
Genome‐wide scan for selective sweeps in the Senegalese sorghum. Selective sweeps in the durra (a) and guinea (b) genomes. Each chromosome was divided into 5,000 grid points each corresponding to one dot. The *y*‐axis represents the composite likelihood ratio (CLR) of each grid point. The vertical dashed lines indicate the colocalized candidate genes with genomic signatures. The horizontal dashed blue line represents the 95th percentile cutoff obtained from 1,000 simulations

Next, we used CLR to identify candidate selective sweeps for Sahelian adaptation in durra in the SSG. Composite likelihood ratio identified 47 candidate genomic regions (top 5% cutoff or CLR > 16.9) in durra (Figure 4a). We investigated if a priori candidate genes (*n* = 64) implicated in stay‐green, flowering time, or inflorescence morphology colocalized with CLR outliers. Given that the candidate genes were identified a priori from the literature, a liberal cutoff of 1 Mb was used to define colocalization between CLR outlier regions and candidate genes. Sixteen out of 47 CLR outliers colocalized with candidate genes (Data S3). The photoperiodic flowering genes *Ma3*, *GI*, *CRY1,* and *ZFL1* and inflorescence architecture candidate genes *HAM3, Sbra2,* and *vt2* colocalized with CLR outliers. The stay‐green loci *Stg3a* and *Stg3b* colocalized with outlier regions on subtelomeric regions of chromosome 2. We used CLR in guinea to identify candidate selective sweeps for Soudanian adaptation. The CLR identified 28 candidate genomic regions (CLR > 10.3) in guinea (Figure 4b). Eleven out of 28 CLR outliers colocalized with candidate genes (Data S3). The photoperiodic flowering genes *PhyA*, *Hd1, SbCN2,* and *Ma6* colocalized with outlier regions. The stay‐green locus *Stg1* colocalized with an outlier region on chromosome 3. The inflorescence morphology genes *IDS1*, *DFL2*, *Sbra3*, and *Dwarf8* colocalized with outlier regions.

### 3.5 Genome‐wide association studies of putative adaptive traits

**Figure 5:**
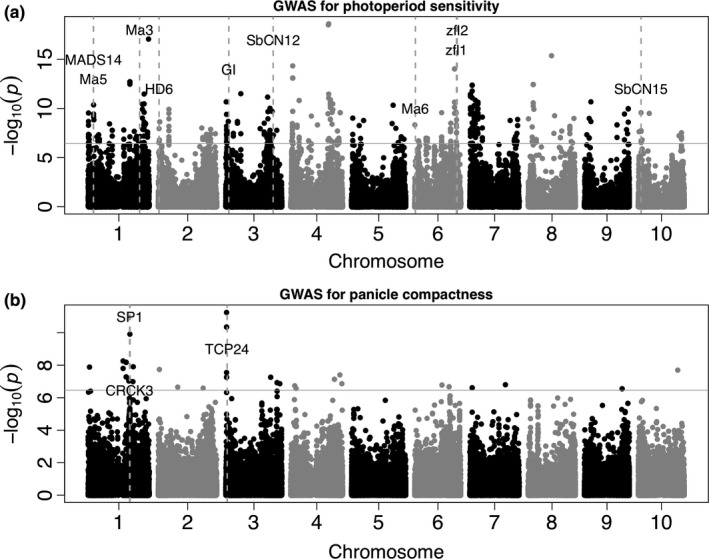
GWAS of photoperiod sensitivity and panicle compactness. Manhattan plots of association tests using the Mixed‐linear model for photoperiod sensitivity (a) and panicle compactness (b) for the whole Senegalese collection. The negative base 10 logarithm of the significance *p*‐value (*y*‐axis) of the SNP‐phenotype association is plotted against the genomic position of each SNP on the chromosomes represented on the *x*‐axis. The gray horizontal line indicates the significance threshold for the Bonferroni corrected *p*‐value > 0.05. Candidate genes colocalizing with significantly associated SNPs are indicated

To better characterize variation underlying putative adaptive traits, we mapped genotype–phenotype associations for photoperiodic flowering and inflorescence morphology. To reduce confounding effects of population structure, we also applied a regional mapping approach where durra accessions were excluded. In total, 445 and 178 significantly associated SNPs (Bonferroni *p*‐value > 0.05) were identified for photoperiod sensitivity for the whole SSG and SSG without durra, respectively (Figure 5a and Figure S5a). Colocalization between associated SNPs and candidate genes was determined based on LD decay rate to background level (*r* ^2^ = 0.1) in durra (800 kb) and guinea (500 kb). Among the associated SNPs, 35 and 26 colocalized with photoperiodic flowering candidate genes for the whole SSG and SSG excluding durra, respectively. For panicle compactness, 48 and 124 significantly associated SNPs were found for the whole SSG and SSG excluding durra, respectively (Figure 5b and Figure S5b). Among the associated SNPs, 13 SNPs colocalized with a priori candidate genes for inflorescence morphology.

Photoperiod sensitivity‐associated SNPs were found near floral regulators *Ma3*, *Ma5*, *Ma6*, *MADS14*, *GI*, *HD6*, *zfl1/2*, *Ehd2*, *SbCN12*, and *SbCN15* (Data S4). Most of these associations were observed whether or not durra were included. The association near *Ehd2* was only observed when durra accessions were excluded, while associations near *Ma6* and *HD6* were only observed when durra accessions were included. Eighteen of the highly significant (*p*‐value > 10^−10^) associations were not near any a priori candidate genes. For panicle compactness, significantly associated SNPs colocalized with *SP1*, *CRCK3*, *TCP24*, *DFL2*, *Sb‐ra2*, *vt2*, and *rel2*. The SNP S1_55302939 (within the *SP1* gene) was significant in both GWAS approaches, while S1_55305415 (1 kb away from *SP1*) was only significant when using the whole SSG panel. Two of the highly significant (*p*‐value > 10^−10^) associations were not near a priori candidate genes.

### 3.6 Environment‐SNP associations

**Figure 6:**
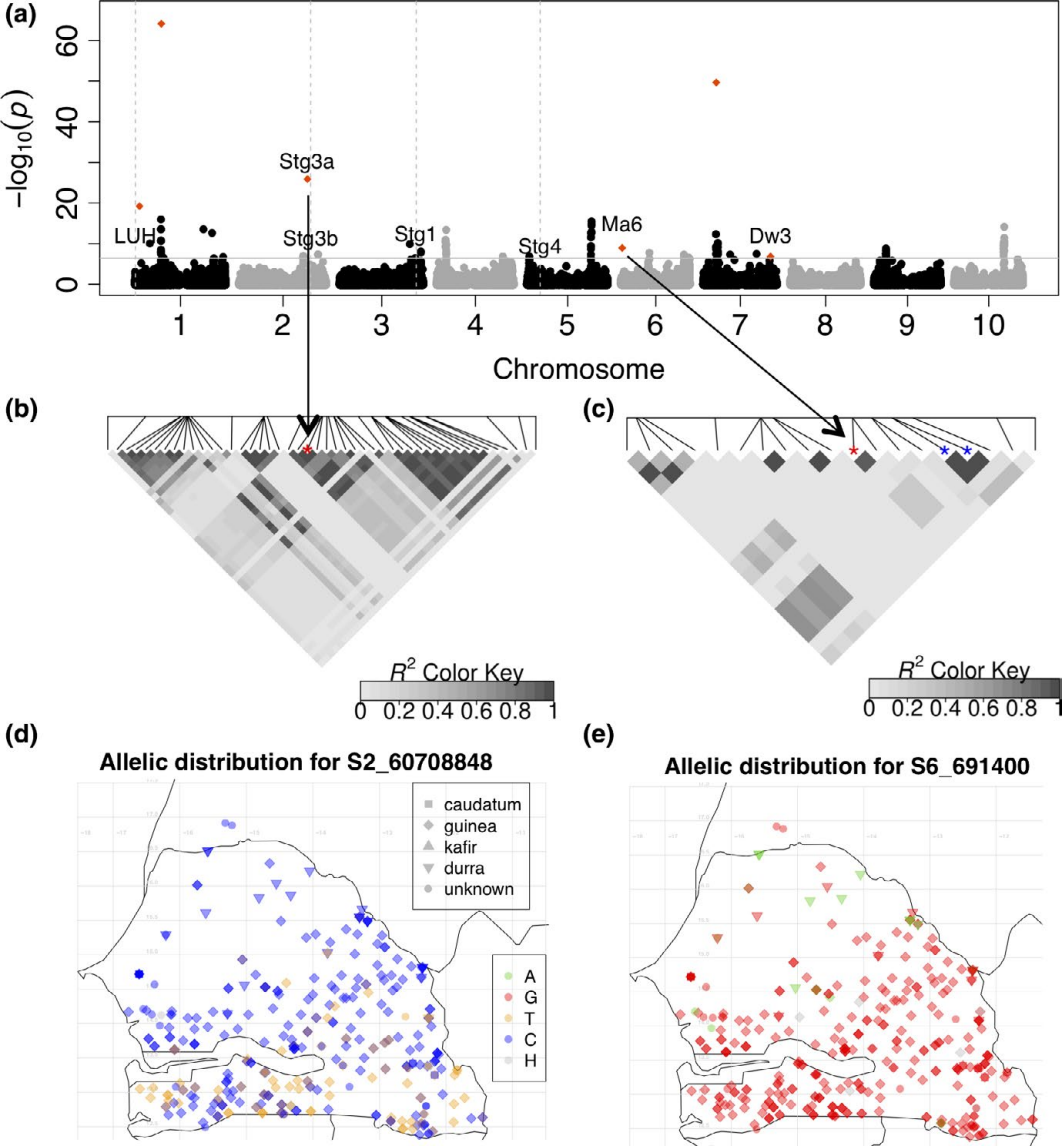
Genome–environment associations for precipitation. (a) SNP associations for “precipitation of the driest quarter” using the generalized‐linear model (GLM). The red dots represent SNPs identified from the multilocus mixed‐model (MLMM). Linkage disequilibrium displayed as heat map of coefficient of correlation *r* ^2^ in a 50 kb region around SNPs S2_60708848 (b) and S6_691400 (c) that colocalize with Stg3a and Ma6 loci in (a), respectively. Red asterisks on each heat map represent these SNPs and blue asterisks indicate the SNPs within Ma6. The color scale indicates the significance of *r* ^2^ values with black color indicating high *r* ^2^ values. Allelic map distribution at SNPs S2_60708848 (d) and S6_691400 (e) associated with precipitation of the driest quarter. The shape of the points indicates the botanical race of the accession and the color indicates the allele at the SNP with H being the heterozygous alleles

We performed GEA to identify SNPs associated with climate variables (Data S4). Based on the GLM, GEA identified 560 SNPs significantly associated (Bonferroni‐adjusted *p*‐value > 0.05) with environment variables including precipitation of the driest quarter (Figure 6a), mean temperature of the warmest quarter (Figure S6a), and precipitation of the wettest quarter (Figure S6b). Associations for longitude variable were based on the MLM (Figure S6c) because GLM identified many associated SNPs. Multilocus mixed‐model identified 16 significantly associated SNPs, including one overlapping SNP (S7_59683060) with the GLM, and 15 additional SNPs that were not identified by GLM or MLM (Table S1, Figure 6a, and Figure S6). Associated SNPs for precipitation of the driest quarter, such as S2_60708848 and S6_691400, identified by the MLMM, colocalized with the *Stg3a* locus and *Ma6* gene, respectively. The stay‐green candidate loci (*Stg1*–*4*) colocalized with SNPs associated with mean temperature of the driest and warmest quarters, precipitation of the driest, warmest and wettest quarters, and longitude (Data S4). The SNP S1_7584419 identified by MLMM as associated with mean temperature of the warmest quarter colocalized with *Ma5* and *MADS14*, but at greater distance (>800 kb).

To determine the pairwise LD between the two SNPs colocalizing with *Stg3a* and *Ma6* and variation within these loci, we generated the LDheatmap of the 50 kb region surrounding each SNP (Figure 6b,c). Nearly complete LD (*r* ^2^ > 0.9) was found between S2_60708848 and other SNPs in the *Stg3a* locus. The SNP S6_691400 was in LD with two SNPs in *Ma6*. The genotypes carrying the minor allele at S2_60708848 were distributed in the southern subhumid environments (Figure 6d). By contrast, genotypes carrying the minor allele at S6_691400 were distributed in the northern and dry environments (Figure 6e). The minor alleles at S3_67831630 (colocalized with *Stg1/SbPIN4*) and S3_57321183 (colocalized with *Stg2/SbPIN2*) were mostly found in durra landraces and few guinea landraces distributed in the dry areas of Senegal (Figure S7a,b).

## 4 DISCUSSION

Genomic analysis of crop landraces can help determine the basis of local adaptation (Lasky et al., 2015; Li et al., 2017; Lin et al., 2014; Swarts et al., 2017). The aims of this study were to characterize factors shaping the genomic variation of Senegalese sorghum landraces, map genomic regions shaped by agroclimatic adaptation, and identify genes that could play a role in local adaptation.

### 4.1 Factors shaping genomic variation of sorghum landraces

Population structure in the Senegalese landraces followed the north–south precipitation gradient. These regional‐scale patterns are in line with global patterns, where population structure is associated with precipitation‐based agroclimatic zones (Lasky et al., 2015). Within Senegalese sorghums, guinea and durra clustered distinctly, consistent with global patterns of genetic differentiation (Morris et al., 2013; Sagnard et al., 2011). The relatively high proportion of variation explained by climate collinear with space suggests a role of clinal adaptation shaping variation, similar to recent findings in Nigerian and global sorghum germplasm (Lasky et al., 2015; Olatoye, Hu, Maina, & Morris, 2018). However, two guinea groups, from the center and south, clustered distinctly (Figure 1b and Figure S2d), suggesting possibly a specific genomic adaptation to the Soudano–Sahelian and the Soudanian agroclimatic, respectively.

The average pairwise nucleotide diversity, observed heterozygosity (data not shown), and the spatial and hierarchical genetic structure observed within guinea group (Figure 2a; File S1) is consistent with guinea being the most genetically diverse sorghum type (Deu et al., 1994; Folkertsma et al., 2005; Morris et al., 2013). Although the number of inferred subpopulations may not always correspond to the number of biological genetic groups (François & Durand, 2010; Meirmans, 2015), the spatial genetic co‐ancestry structure analysis suggests the presence of untapped genetic diversity in the subpopulations in eastern Senegal (Figure 2a). The high estimated admixture coefficients among putative guinea subpopulations (File S1) could be due to gene flow among subpopulations or an effect of limited isolation‐by‐distance. The limited isolation‐by‐distance may occur because the geographic origin of the accessions is not broad (e.g., Senegal is not large) and there is any major geographic barrier that may create isolated subpopulations. There was little evidence of admixture between guinea and durra types, consistent with phenotype studies that rarely identify guinea–durra intermediates (Harlan & De Wet, 1972). Evidence of gene flow was mostly from guinea to durra (e.g., red subgroup at *K* = 7, File S1) and rarely from durra to guinea. The lower abundance of durra in this region may explain the limited gene flow between guinea and durra sorghums.

Ethnicity of farmers has shaped genetic structure in several staple crops including maize and pearl millet (Naino Jika et al., 2017; Orozco‐Ramírez, Ross‐Ibarra, Santacruz‐Varela, & Brush, 2016). Distribution and diffusion of ethnic groups in Senegal including the Wolof, Serer, Diola, and Fulani (Toucouleur, Peul, Peul Foulbe, and Peul Firdou) could have affected gene flow among landraces. Indeed, the ethno‐linguistic origin of the accessions contributed to the genetic variance of the Senegalese sorghum (Figure 2c). Seed exchange among farmers of the same ethnic group may have contributed in shaping this genetic structure (Barnaud, Trigueros, McKey, & Joly, 2008; Orozco‐Ramírez et al., 2016; Pressoir & Berthaud, 2004). Codiffusion of sorghum with human migration has been demonstrated at Africa‐wide scale (Westengen et al., 2014) and at a regional scale in Kenya (Labeyrie, Thomas, Muthamia, & Leclerc, 2016). Durra sorghum in Senegal are grown mainly by the Fulani ethnic group, so the clustering of Senegalese durra with Ethiopian durra (Figure S2d) and low *F*_ST_ (0.052) suggest that durra sorghums moved with Fulani people from northeast Africa (Scheinfeldt, Soi, & Tishkoff, 2010).

### 4.2 Genetic basis of Sahelian and Soudanian adaptation

Nucleotide polymorphism patterns can provide insight into loci underlying adaptation (Vitti, Grossman, & Sabeti, 2013). The reduction of nucleotide polymorphism observed throughout the durra genome (Figure 3a) could be resulted from the bottlenecks during its diffusion along the Sahelian zone. Because Ethiopia is known as the center of origin of durra, we investigated whether the reduced polymorphism in durra was common to all African durra or specific to the Senegalese durra. The results suggest selective sweeps across durra genomes as durra populations diffused along the Sahel (Figure 3c and Figure S4). Interestingly, putative selective sweeps on pericentromeric regions of chromosome 2 were specific to Senegalese durra. By contrast to durra, there was little reduction of nucleotide polymorphism in the guinea genome and predominantly positive values of Tajima's *D* test (Figure 3a,b), reflecting population structure or possible balancing selection (Vitti et al., 2013). Simulations with demographic models could be used for more robust genome scans. Unfortunately, the underlying population parameters (e.g., effective population size, migration rates) are poorly described in sorghum.

Photoperiodic flowering is a key factor underlying adaptation in tropical crops (Kloosterman et al., 2013). The colocalization of photoperiodic flowering candidate genes with putative selective sweeps and phenotypic and environment associations (Figures 4 and 5a; Table S1; Data S4) are consistent with a role of conserved cereal flowering pathways in sorghum climate adaptation. The rare allele at the SNP near *Ma6/Ghd7* (6 kb away) was present in durra genotypes distributed in the drier areas of the Sahelian zone characterized by short growing seasons and low rainfall (<400 mm per year) (Figure 6e). This rare allele may be associated with early maturity and thus suggesting a role in drought escape such that plants can rapidly cover their maturity cycle and produce seeds before the end of growing season.

Other photoperiod flowering regulators identified in U.S. sorghum, *PhyC* (*Ma5*), *PhyB* (*Ma3*), *PhyA*, and *Ma1* (*SbPRR37*), colocalized with phenotype‐associated SNPs (Data S4) and putative selective sweeps in the Senegalese sorghum (Childs et al., 1997; Rooney & Aydin, 1999). Signatures of selection near *Ma3* in durra (*π*_durra_ and CLR; Figure 3a and Figure 4a) are consistent with signatures of positive selection in *Ma3* observed in global sorghum (Wang et al., 2015). The florigens *SbCN12* and *SbCN15* (ortholog of rice florigen *Hd3a*) found near photoperiod‐associated SNPs are photoperiod‐regulated activators of floral induction in U.S. sorghum (Murphy et al., 2011). Putative photoperiodic flowering regulators *SbCRY1* and *SbGI*, colocalizing with selective sweeps in durra and photoperiod‐associated SNPs, were previously associated with photoperiodic flowering in regional West‐Central African germplasm (Bhosale et al., 2012). Several of the above genes were associated with flowering time adaptation in maize landraces, including *PhyB*, *PhyC*, *PRR37*, and *ZFL1/2* (Romero Navarro et al., 2017).

Panicle compactness in sorghum is a function of the number and length of inflorescence branches and the number of aborted spikelets (Brown et al., 2006). Several candidate genes from a previous GWAS of inflorescence branch length in global sorghum (Morris et al., 2013) colocalized with GWAS signals for panicle compactness and/or CLR outliers in the current study (*SP1*, *CRCK3/THE1*, *TCP24*, and *DFL2*). The minor alleles in/near *SP1* (S1_55302939, S1_55305415) observed in durra accessions and some guinea accessions (Figure S7c,d) suggests a rare variant in *SP1* could contribute to shorter inflorescence branches in some Senegalese sorghum.

The colocalization of selective sweeps and GEA (Data S4) with stay‐green drought tolerance loci (Borrell et al., 2014) suggests a broader role for stay‐green loci in Sahelian adaptation. A selective sweep and associated SNPs colocalized with the stay‐green locus *Stg1*/*SbPIN4* in guinea sorghums, suggesting that this region may confer adaptation of some guinea accessions to the dry areas of Senegal. The rare allele of SNP S3_57321183, which colocalized with *SbPIN2,* was found in a few guinea sorghums (Figure S7b). One possibility is that severe droughts starting in the 1970s (Gautier, Denis, & Locatelli, 2016; Mbow, Mertz, Diouf, Rasmussen, & Reenberg, 2008) have favored the introgression of stay‐green drought tolerance alleles into some guinea landraces. Genome scans comparing older landrace collections with recent collections may shed more light on whether more recent selection (e.g. 1970s–2000s) has occurred, as demonstrated in Sahelian pearl millet (Vigouroux et al., 2011).

### 4.3 Prospects for genomic dissection and improvement of climate adaptation

Improving adaptation of staple crops to the Sahelian and Soudanian zones is critical for smallholder farmers and a major challenge for African plant breeders. Despite advances in genotyping platforms, genomic tools for crop adaptation in sub‐Saharan countries remain lacking. This study generated substantial genomic resources (213,916 SNPs among which 145,235 SNPs have MAF > 0.05) representing high‐quality markers useful for the genomic dissection of adaptive and complex traits. High rates of SNPs with low frequency minor alleles (about 60% of the data had MAF < 0.05) were detected. One possible explanation may be related to the fact that these accessions are mostly landraces grown in their center of origin; thus high number of rare polymorphisms might be segregating at intermediate frequency in the germplasm. In the USDA‐NPGS Ethiopian sorghum collection, similar patterns of MAF were found where 60% of detected SNPs had MAF < 0.05 (Cuevas, Rosa‐Valentin, Hayes, Rooney, & Hoffmann, 2017). Overall, the Senegalese sorghum landraces represent a useful genetic resource, harboring useful variation for maturity and inflorescence morphology, as well as resistant sources to grain mold and anthracnose (Cuevas, Prom, & Rosa‐Valentin, 2018).

The moderate decay of LD observed within the germplasm (Figure S2b) is consistent with the predominance of inbreeding in sorghum (Hamblin et al., 2005). Studies in sorghum have found a comparable LD pattern, decaying to its background level at ~150 kb (Mace et al., 2013; Morris et al., 2013). The population structure of the Senegalese sorghum landraces would be expected to increase spurious association and reduce the power of GWAS (Brachi, Morris, & Borevitz, 2011). Indeed, the number of associations for photoperiod sensitivity was reduced when applying the regional mapping approach excluding durra accessions, presumably due to fewer spurious associations. Future studies with West African multi‐parent mapping populations could breakup confounding LD and improve power to detect climate‐adaptive loci (Bouchet et al., 2017; McMullen et al., 2009).

The stay‐green loci may be useful to improve for drought adaptation in the Sahel via marker‐assisted selection. Circadian clock‐related genes influence crop yield under abiotic stress (Bendix, Marshall, & Harmon, 2015) and photoperiodic flowering loci identified may contribute to early maturity and drought escape in the Sahel. Taken together, our findings suggest a complex oligogenic basis of adaptation to Sahelian versus Soudanian climate, underpinned by variation in conserved floral regulatory pathways and variation in other pathways that are more poorly understood. Whole‐genome resequencing of African crop diversity for GWAS and genome scans could facilitate identification of causal variants in the molecular pathways that underlie climate adaptation.

## CONFLICT OF INTEREST

None declared.

## AUTHOR CONTRIBUTIONS

G.M., D.F., and N.C. conceived and managed the study. F.M., J.F, and Z.H. generated the SNP data set. J.F. analyzed the data. J.F. and G.M. wrote the manuscript.

## Data Availability

The raw sequencing data generated in this study are available in National Center for Biotechnology Information under the BioProject accession number PRJNA433571. The SNP data set is available at Dryad Data Repository under accession https://doi.org/10.5061/dryad.32f5395.
