# Supplementary material for: Genomic signatures of adaptation to Sahelian and Soudanian climates in sorghum landraces of Senegal
Source: Ecol Evol. 2019 Apr 23;9(10):6038–51. doi: 10.1002/ece3.5187 (PMC6540697; doi:10.1002/ece3.5187)

Supporting Information File S1 Genetic admixture of the SSG sorghums based on ADMIXTURE model-based clustering using unlinked 129,460 SNPs markers. Hierarchical genetic clustering in the Senegalese sorghum collection based on Bayesian posterior probability of membership at k = 3, 4, 5, 6 and 7 subgroups. Each vertical bar on the x-axis corresponds to one accession whose proportion of membership in different subgroups is represented by a given k color. The color-coding of the main panels is random, but that of the rugged bars corresponds to the different morphological types [e.g. guinea (orange), durra (green), caudatum (blue), bicolor (red), guinea-caudatum (sky blue)], and unknown (gray). Accession names and origins are indicated from left to right, respectively at the left hand side of each bar plot. The significant gene flow from guinea to durra can be observed in the red subgroup at k = 7.

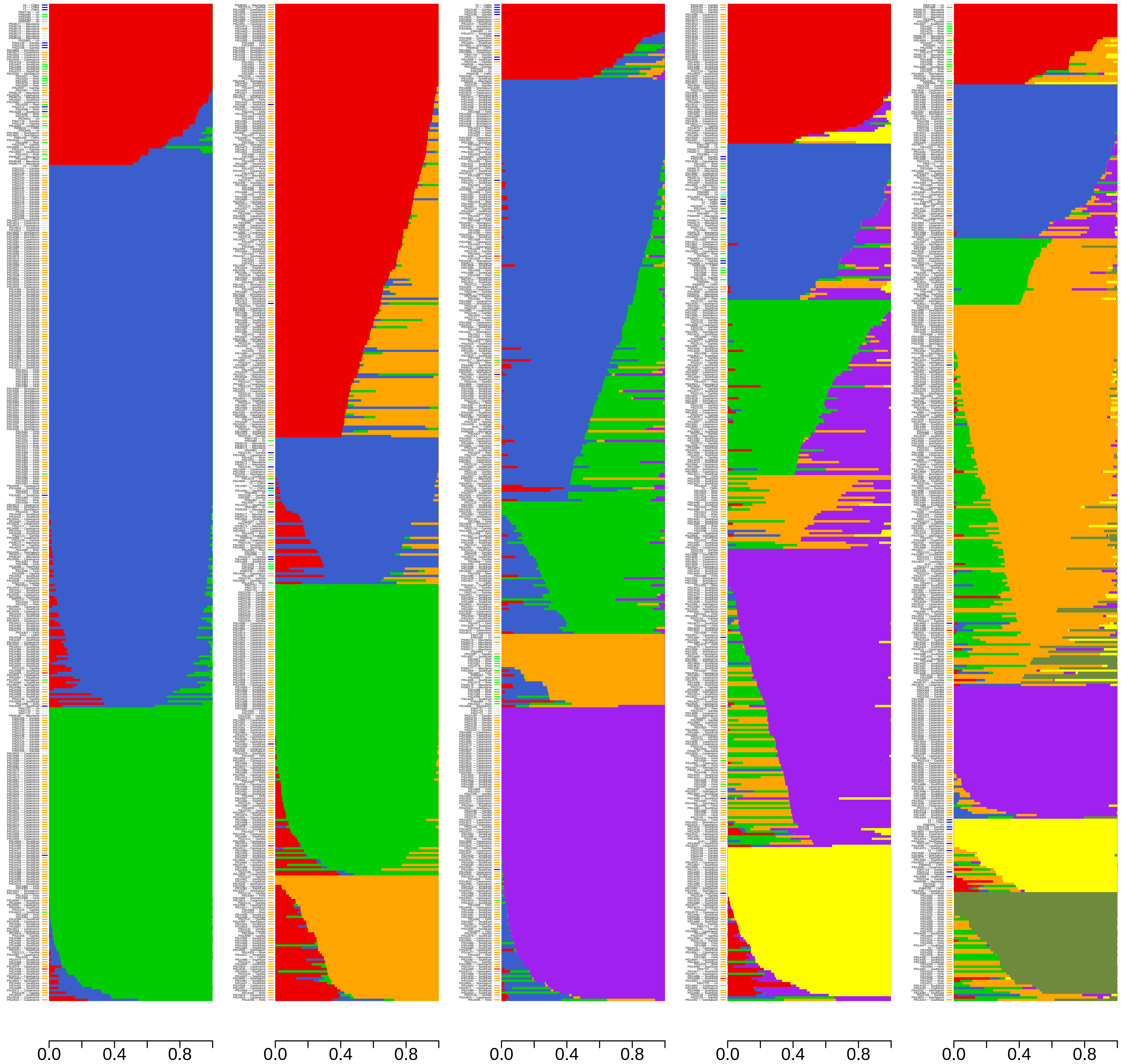

Supplement: Supplementary file 5 [file ECE3-9-6038-s005.pdf]
