# Supplementary material for: Genomic signatures of adaptation to Sahelian and Soudanian climates in sorghum landraces of Senegal
Source: Ecol Evol. 2019 Apr 23;9(10):6038–51. doi: 10.1002/ece3.5187 (PMC6540697; doi:10.1002/ece3.5187)
Supplement: Supplementary file 6 [file ECE3-9-6038-s006.docx]

**Supporting Information for:**

**Genomic signatures of adaptation to Sahelian and Soudanian climates in sorghum landraces of Senegal**

Jacques M. Faye, Fanna Maina, Zhenbin Hu, Daniel Fonceka, Ndiaga Cisse, Geoffrey P. Morris^*^

Author for correspondence:

*Geoffrey P. Morris*

*Tel: 1-785-532-3397*

*Email: gpmorris@ksu.edu*

**Supporting Information Fig. S1**

Map of Senegalese accessions distribution colored-coded with respect to geographic region of origin (**A**) and ethno-linguistic groups (**B**). The color background scale indicates the annual precipitation in millimeters with green color representing the highest precipitation of the Soudanian zone, pink representing lowest precipitation of the Sahelian zone, and yellow representing the zone of transition between Sahelian and Soudanian zones. The improved varieties (yellow) were assigned to the coordinates of the Centre National de Recherche Agronomic (CNRA) of Bambey, where they were developed. Casa corresponds to the region of Casamance, P_Firdou and P_Foulbe correspond to Peul Firdou and Peul Foulbe, respectively.


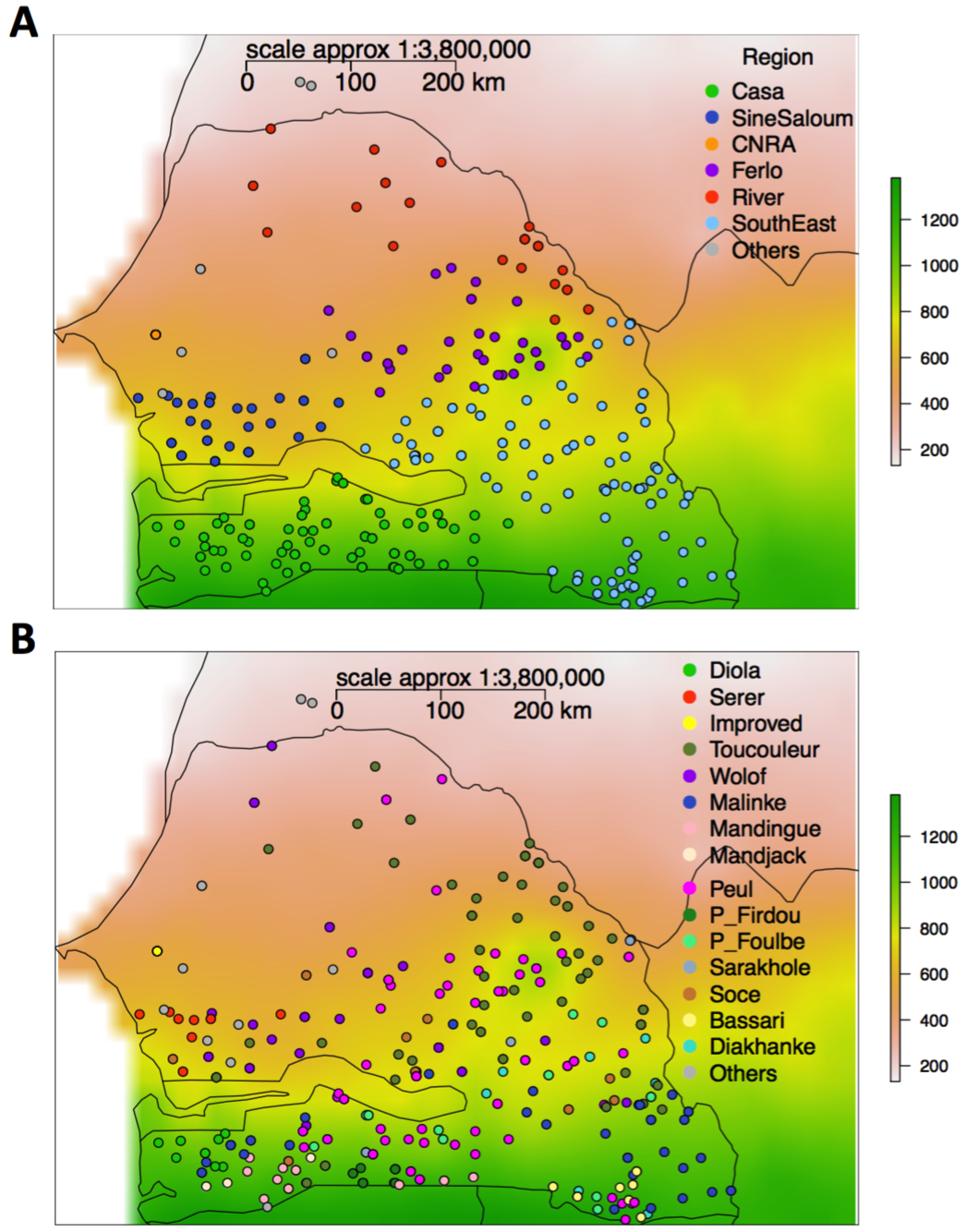


**Supporting Information Fig. S2**

Nucleotide polymorphisms variation and relationship between the Senegalese sorghum landraces and worldwide sorghums. (**A**) SNP markers (213,916 SNPs, MAF > 1%) distribution across the 10 sorghum chromosomes of the Senegalese sorghum landraces in the USDA Germplasm Resources Information Network genebank (SSG). SNP marker density was determined based on non-overlapping window size of 1 Mb. (**B**) Linkage disequilibrium decay along the genome in the whole SSG, in guinea accessions, and in durra accessions within the SSG. (**C**) Distribution of minor allele frequencies for the SNP data sets across the 421 accessions in the SSG (red) and 580 accessions in the global sorghum diversity panel (GDP) (blue). (**D**) Neighbor-joining tree based on genetic similarities between accessions of the SSG (red dots), other West African accessions (magenta dots), Ethiopian accessions (cyan dots), and the GDP (gray dots). The botanical races are represented by edges of the tree.


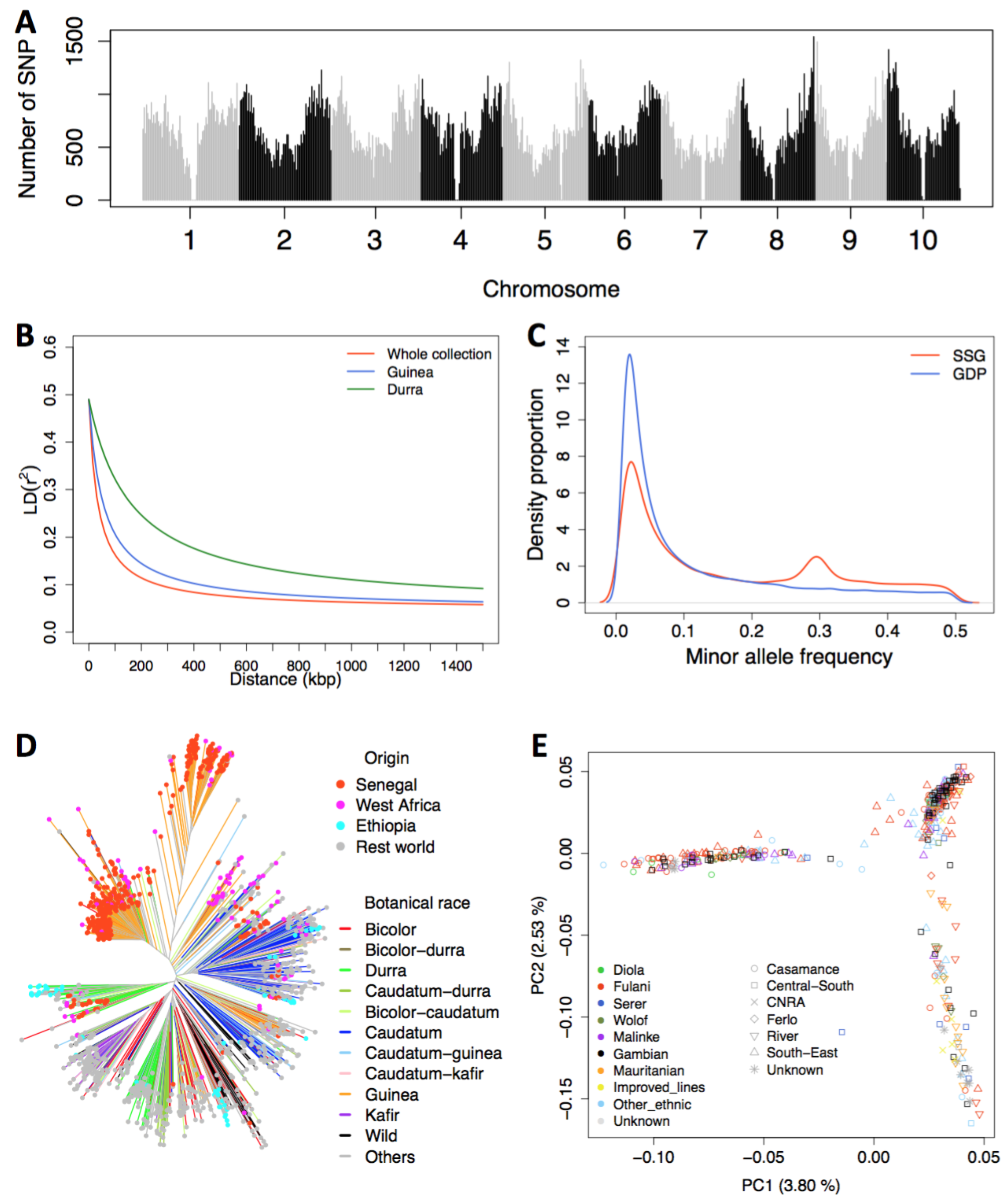


**Supporting Information Fig. S3**

Cross validation error of the model-based clustering of ADMIXTURE program. The optimum number of subpopulations corresponded to K = 7.


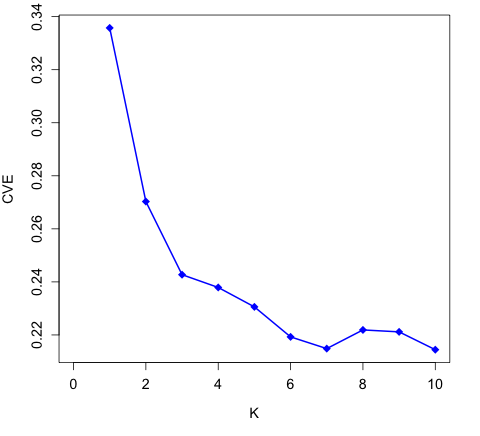


**Supporting Information Fig. S4**

Decreased pairwise nucleotide diversity in durra sorghums along the Sahel. Signatures of positive selection between all guineas in the GDP and Ethiopian durra (blue), West African durra–Niger and Mali (Green), and Senegalese durra in the SSG (red). The horizontal dashed lines indicate the mean value (blue) and the top 5% (gray) of the decreased nucleotide diversity based on non-overlapping sliding windows of 1Mbp.


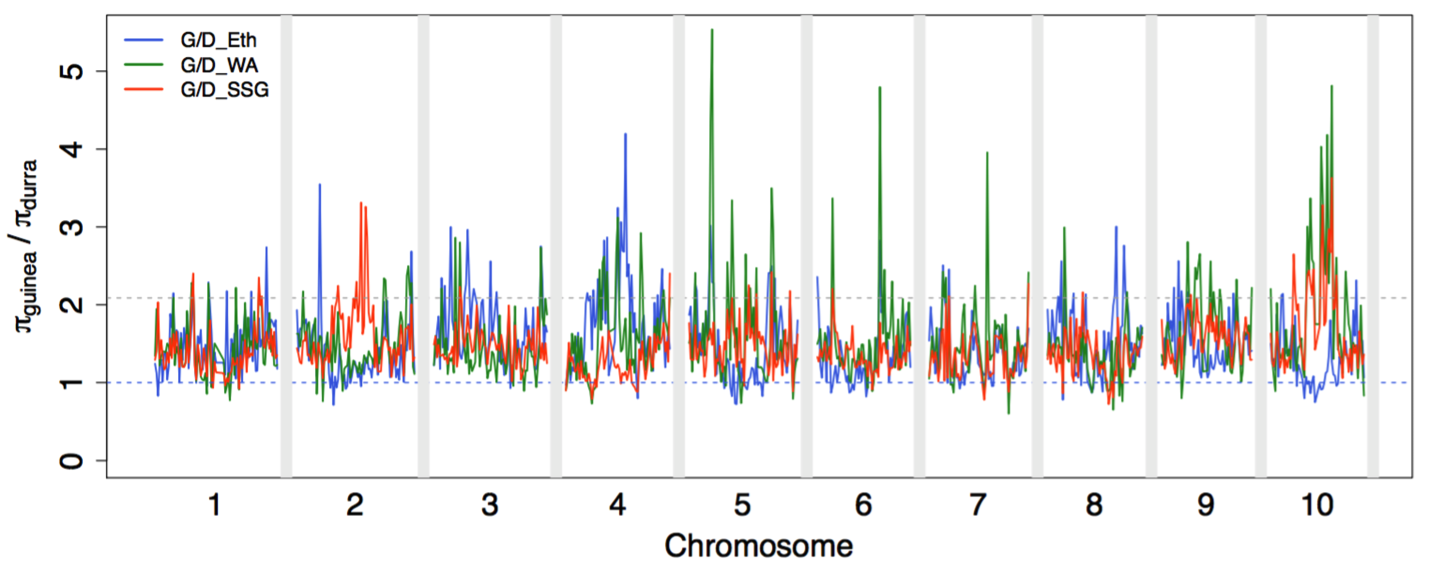


**Supporting Information Fig. S5**

Manhattan plots for the regional mapping GWAS, where durra sorghums accessions were excluded, using the Mixed-linear model for photoperiod sensitivity (**A**) and panicle compactness (**B**) for the SSG sorghum landraces. The negative base 10 logarithm of the significance *p*-value (y-axis) of the SNP-phenotype association is plotted against the genomic position of each SNP on the ten chromosomes (x-axis). The gray horizontal line indicates the significance threshold for the Bonferroni corrected *p*-value > 0.05. Candidate genes co-localizing with significantly associated SNPs are indicated.


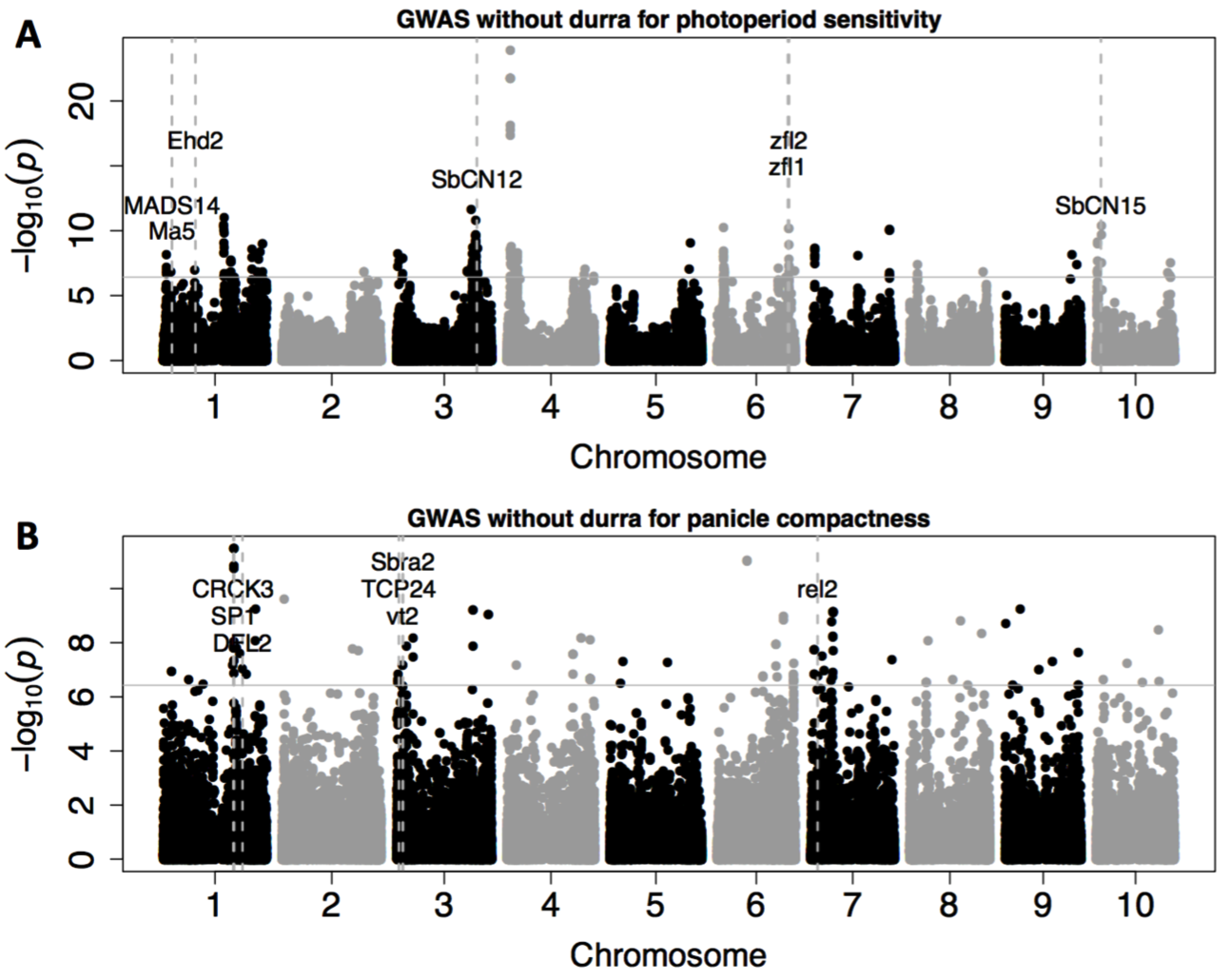


**Supporting Information Fig. S6**

Genotype-environment associations for adaptation in the Senegalese sorghum landraces. (**A**) Manhattan plot showing SNPs associated with mean temperature of the warmest quarter using the generalized-linear model (GLM). (**B**) SNPs associated with precipitation of the wettest quarter using GLM. (**C**) Manhattan plot showing SNPs associated with longitude using the mixed-linear model (MLM). The GLM identified many associations for longitude variable, so the MLM results are showed here. The red dots on each plot represent significantly associated SNPs identified from the multi-locus mixed-model (MLMM). The x-axis represents the SNP position on the ten chromosomes of sorghum. The y-axis indicates the significance of the associations.


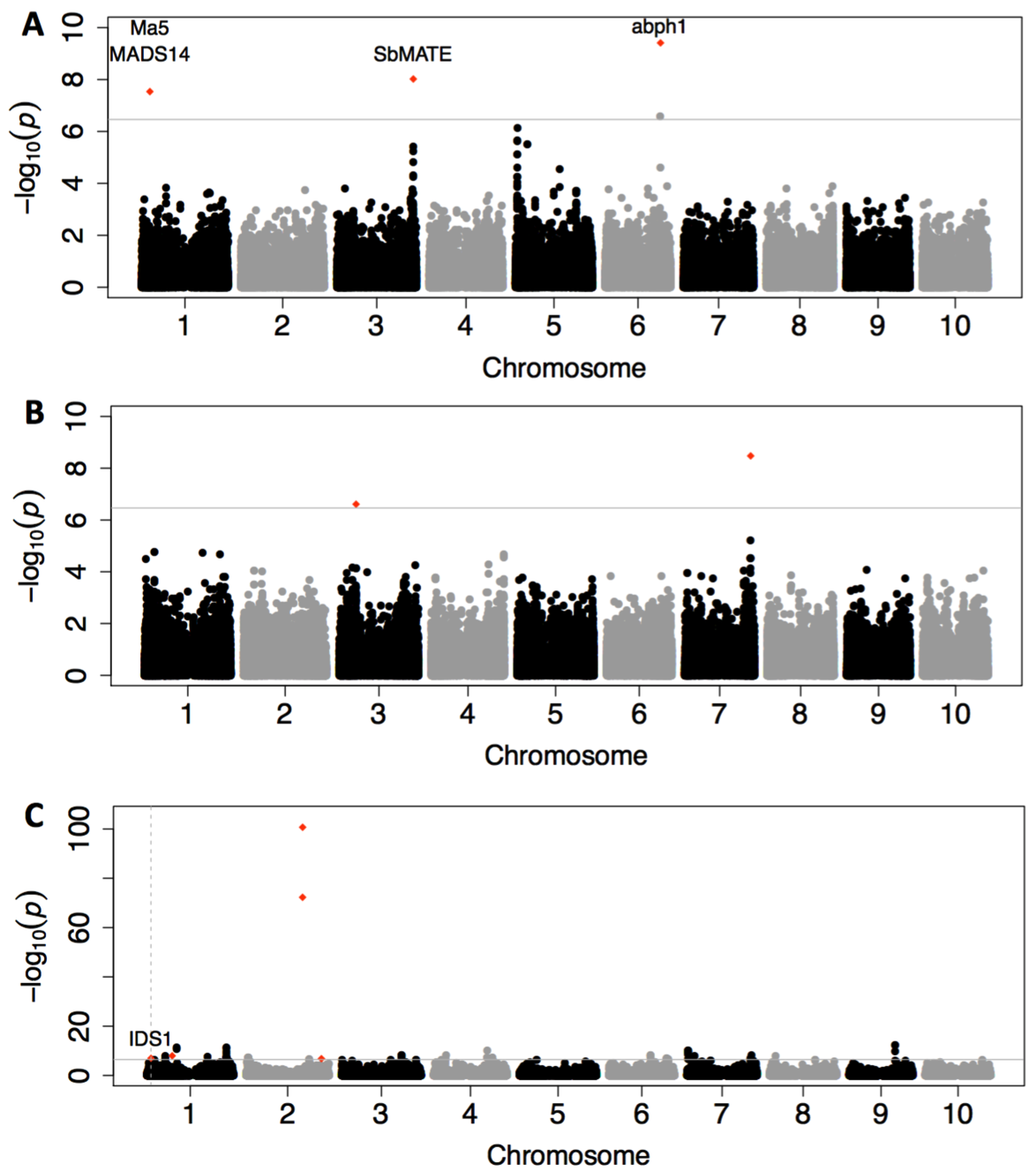


**Supporting Information** **Fig. S7**

(**A**) Allelic map distribution at SNP S3_67831630 significantly associated with precipitation of the driest quarter and co-localizing with *Stg1/SbPIN4* locus. The minor allele at this SNP is found in durra landraces distributed in the dry areas in the Sahelian zone of Senegal. (**B**) Allelic map distribution at SNP S3_57321183 associated with precipitation of the wettest quarter and co-localizing with *Stg2/SbPIN2* locus. The minor allele at this SNP is mostly found in durra and a few guinea accessions of in the drier areas of Senegal. Allelic map distribution at SNPs S1_55302939 (**C**) and S1_55305415 (**D**) associated with panicle compactness and co-localizing with the *SP1* candidate gene*.*


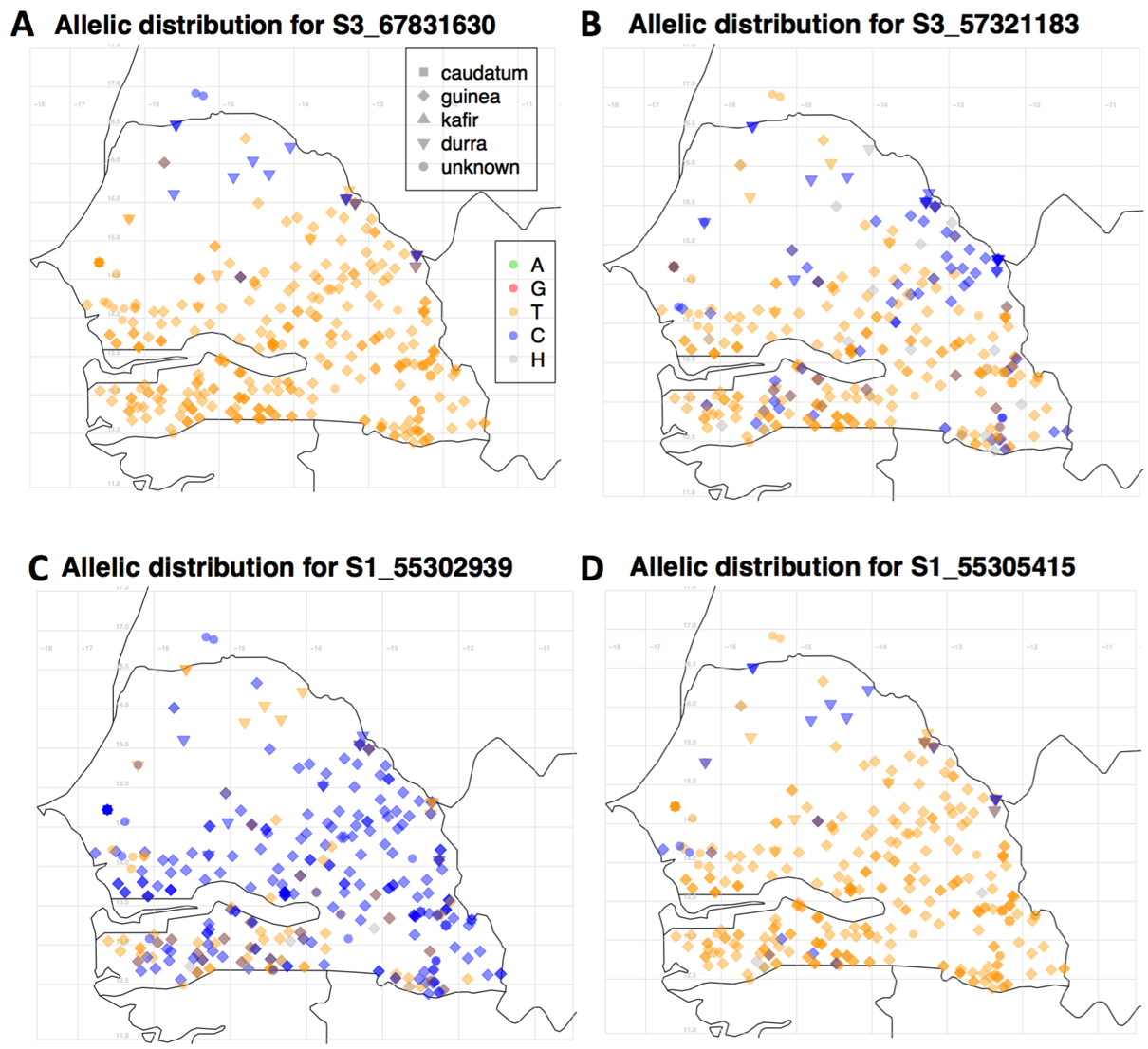


**Supporting Information Table S1**: Environment-SNP associations identified by the multi-locus mixed-linear model (MLMM). In the SNP column, the digit after “S” indicates the chromosome number and the other digits after the underscore indicate the SNP position.

SNP P-value MAF Closest gene/locus Position to gene (kb)

*Precipitation of the driest quarter*

S1_23271724 7.87E-65 0.287323944

S7_11301809 2.23E-50 0.270422535

S2_60708848 1.25E-26 0.281690141 *Stg3a* within

S1_3836265 5.85E-20 0.285915493

S6_691400 1.08E-09 0.073239437 *Ma6* 6

S7_59683060 1.40E-07 0.050704225 *Dw3* 138

*Mean temperature of the warmest quarter*

S1_7584419 2.92E-08 0.090140845 MADS14; *Ma5* 884; 830

S6_51709806 3.89E-10 0.243661972 *abph1* 454

S3_71370900 9.54E-09 0.173239437 *SbMATE* 262

*Precipitation of the wettest quarter*

S7_61856992 3.34E-09 0.091549296

S3_15688290 2.46E-07 0.333802817

*Longitude*

S2_52993994 2.05E-101 0.057746479

S2_52937592 5.40E-73 0.057746479

S1_23473920 1.07E-08 0.290140845

S1_3547581 1.11E-07 0.057746479 *IDS1* 806

S2_70618491 1.56E-07 0.285915493

______________________________________________________________________________
